# Impact of *BCR::ABL1* single nucleotide variants on asciminib efficacy

**DOI:** 10.1038/s41375-024-02411-7

**Published:** 2024-09-17

**Authors:** Andrew J. Innes, Chloe Hayden, Victoria Orovboni, Simone Claudiani, Fiona Fernando, Afzal Khan, David Rees, Jennifer Byrne, Paolo Gallipoli, Sebastian Francis, Mhairi Copland, Gillian Horne, Manoj Raghavan, Claire Arnold, Angela Collins, Tanya Cranfield, Nicholas Cunningham, Akila Danga, Peter Forsyth, Rebecca Frewin, Paula Garland, Guy Hannah, Daniele Avenoso, Sandra Hassan, Brian J. P. Huntly, Jissan Husain, Sudhakaran Makkuni, Kate Rothwell, Jamshid Khorashad, Jane F. Apperley, Dragana Milojkovic

**Affiliations:** 1 Centre for Haematology, Faculty of Medicine, Imperial College London, London, United Kingdom; 2 Department of Haematology, Hammersmith Hospital, Imperial College Healthcare NHS Trust, London, United Kingdom; 3 North West London Pathology, Hammersmith Hospital, Imperial College Healthcare NHS Trust, London, United Kingdom; 4 Medical School, Faculty of Medicine, Imperial College London, London, United Kingdom; 5 Centre for Clinical Haematology, Nottingham University Hospitals NHS Trust, Nottingham, United Kingdom; 6 Centre for Haemato-Oncology, Barts Cancer Institute, Queen Mary University of London, London, United Kingdom; 7 Department of haematology, Sheffield Teaching Hospitals NHS Trust, Sheffield, United Kingdom; 8 Paul O’Gorman Leukaemia Research Centre, School of Cancer Sciences, University of Glasgow, Glasgow, United Kingdom; 9 Centre for Clinical Haematology, Queen Elizabeth Hospital, Birmingham, United Kingdom; 10 Department of Haematology, Belfast City Hospital, Belfast, United Kingdom; 11 Department of Haematology, Norfolk and Norwich University Hospital, Norwich, United Kingdom; 12 Department of Haematology, Queen Alexandra Hospital, Portsmouth, United Kingdom; 13 Department of Haematology, The Hillingdon Hospital, London, United Kingdom; 14 Department of Haematology, Raigmore Hospital, NHS Highland, Inverness, United Kingdom; 15 Department of Haematology, Gloucestershire Royal Hospital, Gloucester, United Kingdom; 16 Department of Haematology, Princess Royal University Hospital, London, United Kingdom; 17 Department of Haematology, Kings College Hospital, London, United Kingdom; 18 Department of Haematology, Queen’s Hospital, Romford, United Kingdom; 19 Wellcome-MRC Cambridge Stem Cell Institute, University of Cambridge, Cambridge, United Kingdom; 20 Department of Haematology, Ashford and St Peter’s Hospitals NHS Foundation Trust, Chertsey, United Kingdom; 21 Department of Haematology, Mid and South Essex NHS Foundation Trust, Basildon, United Kingdom; 22 Department of Haematology, Leeds Teaching Hospitals NHS Trust, Basildon, United Kingdom; 23 Haemato-oncology Molecular Diagnostic Unit, The Royal Marsden Hospital NHS Foundation Trust, Sutton, United Kingdom

**Keywords:** Chronic myeloid leukaemia, Targeted therapies

## Abstract

Asciminib is a potent and selective inhibitor of BCR::ABL1, with potential to avoid toxicity resulting from off-target kinase inhibition. Forty-nine patients treated with asciminib under a managed access program in the UK were evaluated for toxicity and response. Intolerance, rather than resistance (65% vs. 35%), was the most common reason for cessation of the last-line of treatment but asciminib was well tolerated, with most patients (29, 59%) remaining on treatment at a median of 14 months follow-up, and only 6 (12%) stopping for intolerance. Of 44 patients assessable for response, 29 (66%) achieved a complete cytogenetic response (CCyR) or better, with poorer responses seen in those stopping their last-line of therapy for resistance. Fewer patients with a prior history of a non-T315I-*BCR::ABL1* single nucleotide variant (BSNV), or a non-T315I-BSNV detectable at baseline achieved CCyR. Serial tracking of BSNV by next generation sequencing demonstrated clonal expansion of BSNV-harbouring populations, which in some settings was associated with resistance (E459K, F317L, F359I), while in others was seen in the context of ongoing response, often with intensified dosing (T315I, I502F). These data suggest that asciminib exerts selective pressure on some BSNV-harbouring populations in vivo, some of which may respond to intensified dosing.

## Introduction

Asciminib is an allosteric BCR::ABL1 inhibitor that binds the myristoyl pocket of ABL1. In contrast to catalytic-site ABL1 kinase inhibitors that block ATP-binding, asciminib induces a conformational change in the BCR::ABL1 resulting in inactivation of the kinase function [1]. While the development of ATP-competitive tyrosine kinase inhibitors (TKIs) has transformed the treatment landscape for patients with chronic myeloid leukaemia (CML), leading to near-normal life expectancy [2], this is not ubiquitous. A significant proportion of patients remain resistant to conventional TKIs, or experience toxicity that is either dose-limiting, or results in long term morbidity [3], with many of the TKI-associated toxicities deriving from their off-target kinase inhibition, e.g. src or VEGF [4–6]. Importantly, myristoyl-binding sites analogous to that of ABL1 are only present in a very limited number of kinases, leading to a higher degree of specificity of asciminib for the BCR::ABL1 oncoprotein [7] that in turn predicts for fewer off-target toxicities. This, coupled with a high potency for BCR::ABL1, implies that asciminib should be both effective and well tolerated, and data from clinical trials with pre-treated CML patients are encouraging [8, 9]. Nevertheless, not all patients respond to asciminib, and while the impact of tyrosine kinase domain (TKD) mutations in resistance to conventional TKIs is well established [10], their impact on asciminib efficacy is less clear. Additionally, because domains other than the TKD are necessary for BCR::ABL1 inhibition by asciminib, variant screening must be extended. Given that clinically relevant variants are no longer restricted to the TKD only, they are herein termed *BCR::ABL1* single nucleotide variants (BSNV).

Early optimism that the BSNV resistance-spectrum of conventional TKIs may have little or no impact on the efficacy of asciminib has not borne out in practice, but the degree of spectral overlap requires further investigation. Of note, in vitro assays show that while some BSNV increase the IC50 of asciminib [1], the doses required for inhibition remain only modestly increased [1, 11, 12] and may still be within a deliverable therapeutic window. This is most well described in the setting of the T315I-BSNV, where the in vitro IC50 of asciminib is around 8 to 12-fold that of non-mutated BCR::ABL1, in comparison to 100 to 1000-fold for imatinib, bosutinib, dasatinib and nilotinib [1, 11], and where robust clinical responses are seen with escalated asciminib dosing schedules [8, 13]. The degree to which other BSNV result in asciminib resistance, and whether these can also be overcome with escalated dosing is unknown.

In this study we have collated response and toxicity data from UK patients receiving asciminib on a managed access programme from Novartis. We have particularly focused on toxicity and factors associated with response, and have used targeted next generation sequencing (NGS) of the *BCR::ABL1* fusion sequence, which affords higher sensitivity and permits monitoring of variant allele frequency (VAF) [14, 15] to better understand the impact of BSNV on asciminib response.

## Methods

### Study overview

This retrospective cohort study gathered data from 14 centres across the UK. The study was approved by a Local Research Ethics Committee. All patients received asciminib provided by Novartis through a managed access programme provided the following criteria were met: (1) treatment need of a serious or life-threatening disease lacking commercially available options; and (2) the patient being ineligible or unable to participate in a clinical trial. The recommended standard dose was 40 mg twice daily, with escalated doses of 200 mg twice daily for those with a history of a T315I-BSNV. With the exception of one patient with failed engraftment and full autologous reconstitution more than 20 years ago, those with prior history of allogeneic hematopoietic stem cell transplantation were excluded from this analysis, and have been previously reported [16].

*BCR::ABL1* levels were expressed as the *BCR::ABL1/ABL1* ratio on the international scale for patients with e13a2 and/or e14a2 transcripts, and as *BCR::ABL1/ABL1* ratios from a single laboratory for one patient with an e19a2 transcript. Response was assessed according to the 2020 ELN criteria, and 2023 ELN laboratory criteria [17, 18] with complete cytogenetic response (CCyR) defined as *BCR::ABL1* IS level ≤1%, major molecular response (MMR) ≤ 0.1%, and deep molecular response, MR4 ≤ 0.01%, and MR4.5 ≤ 0.0032%. The single patient with an e19a2 transcript had no significant reduction in transcript level on asciminib (lowest *BCR::ABL1/ABL1* PCR of 18% after 12 months of treatment), so was deemed not to have achieved a CCyR for the purposes of response assessment. Treatment emergent adverse events (TEAEs) were graded according to the National Cancer Institute Common Terminology Criteria for Adverse Events Version 5.0 where possible.

BSNV analysis was performed after amplification of *BCR::ABL1* as described in the supplemental methods, in line with previous methodology [15]. Sequences were aligned against *ABL1* reference sequence NM_005157.6, with analysis of amino acid positions 220–509. Synonymous variants and single nucleotide polymorphisms (SNPs) were excluded from analysis. The limit of detection for this methodology was established at a VAF of 3%, but variants detected <3% that were reproducible across independent NGS runs were included in the analysis.

Data analysis was performed with SPSS Version 26.0. Continuous variables are reported as median values and compared by the Mann-Whitney-U test, and categorical variables were compared by 2-sided Chi-squared.

## Results

### Patient Cohort

Forty-nine patients were included in this analysis with the full demographics shown in Table 1, and the analysis workflow shown in the consort diagram (supplementary Fig. 1). Briefly, this was a heavily pre-treated cohort (median prior lines of TKI was 4 [2-5]), with the majority having received prior ponatinib (*n* = 29 (59%)), of whom 9 (18%) had been resistant. Most (*n* = 35, 71%) had achieved CCyR or better to at least one line of prior therapy, and the reason for discontinuation of the last therapy was intolerance in 32 (65%) and resistance in 17 (35%).

**Table 1:** Cohort demographics (*n* = 49).

Characteristic		Number (%)/Median [Range]
**Age at diagnosis (years)**		53.5 [14–80]
**Age at Asciminib (years)**		59 [23–88]
**Gender**		
	Male	27 (55%)
	Female	22 (45%)
**Disease phase at diagnosis**		
	Chronic	48 (98%)
	Accelerated	1 (2%)
**Disease phase at asciminib initiation**		
	Chronic	49 (100%)
**ACA at diagnosis**		
	No	36 (73%)
	Yes	3 (6%)
	Unknown	10 (20%)
**ELTS score at diagnosis**		
	Low	16 (33%)
	Intermediate	9 (18%)
	High	4 (8%)
	Missing	20 (41%)
**Transcript type**		
	e13a2	19 (39%)
	e14a2	12 (24%)
	e13a2/e14a2	9 (18%)
	Unknown, (non-rare)	8 (16%)
	e19a2	1 (2%)
**Time since diagnosis (months)**		73 [11–386]
**Number of prior TKIs (median)**		4 [2–5]
**Last TKI**		
	Imatinib	1 (2%)
	Bosutinib	17 (35%)
	Dasatinib	5 (10%)
	Nilotinib	4 (8%)
	Ponatinib	22 (45%)
**Prior ponatinib use**		29 (59%)
**Ponatinib resistant**		9 (18%)
**Reason for stopping last TKI**		
	Resistance	17 (35%)
	Intolerance	32 (65%)
**Achieved CCyR to at least 1 previous line of therapy**		35 (71%)
**BSNV (History)**		
	Any	21 (43%)
	T315I	11 (22%)
	Non-T315I-BSNV all	12 (24%)
	Non-T315I-BSNV clinically significant	10 (20%)

*ACA* additional chromosomal abnormalities, *ELTS* Eutos long-term survival, *TKI* tyrosine kinase inhibitor, *CCyR* complete cytogenetic response, *BSNV*, *BCR::ABL1* single nucleotide variants.

A significant proportion (*n* = 21, 43%) had a history of at least one known BSNV, and 11 (22%) had a history of a T315I-BSNV (Supplementary Tables 1 and 2). The median maximum tolerated dose of asciminib in patients with a history of a T315I-BSNV was 400 mg [80–400] daily and 80 mg [20–400] daily for those without a T315I-BSNV history (*P* < 0.001).

Comorbidities were common, with 60% of patients having a history of at least one cardiovascular condition defined by the presence of hypertension, peripheral vascular disease (PVD), ischaemic heart disease (IHD), atrial fibrillation (AF), stroke or transient ischaemic attack (TIA). Hypertension was the most frequent, seen in 18 (37%) patients, with PVD and IHD in 9 (18%) patients each, AF in 8 (16%) and stroke or TIA in 4 (8%). Diabetes was present in 4 (8%) patients, and chronic kidney disease in 9 (18%).

**Fig. 1:**
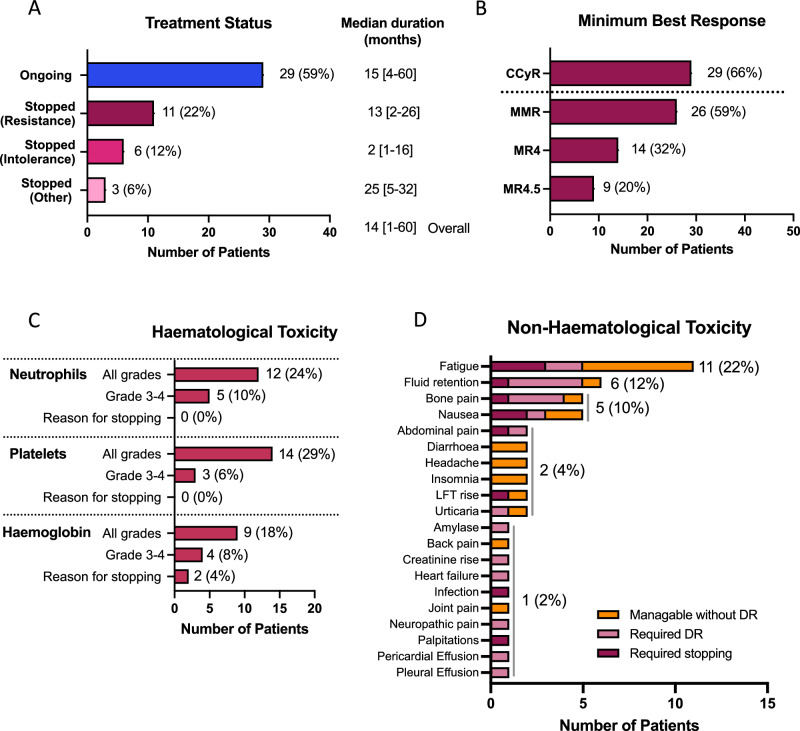
Clinical outcome and toxicity data. **A** Treatment status at time of data collection, **B** best response achieved on asciminib, **C** haematological toxicity and **D** non-haematological toxicity. CCyR: complete cytogenetic response (*BCR::ABL1* IS level ≤ 1%), MMR, major molecular response (*BCR::ABL1* IS level ≤ 0.1%), MR4, *BCR::ABL1* IS level ≤ 0.01% IS, MR4.5, *BCR::ABL1* IS level ≤ 0.0032% IS, LFT: liver function tests, DR: dose reduction, IS: international scale.

The median duration of asciminib treatment for the entire cohort was 14 [1–60] months. At the time of data reporting, 29 (59%) patients remained on treatment with asciminib (Fig. 1A), with median duration of 15 [4–60] months, while 11 (22%) had stopped for resistance with a median duration of treatment of 13 [2–26] months, and 6 (12%) for intolerance with a much shorter median duration of 2 [1 to 16] months before stopping. Three (6%) stopped for other reasons; one each for treatment-free remission attempt, poor compliance and attempting pregnancy with a median duration of treatment of 25 [5–32] months.

### Toxicity

All 49 patients were evaluated for toxicity. Haematological toxicity of any grade was seen in 19 (39%) patients, with grade 3–4 in 9 (18%). Thrombocytopenia was most common (all grades, 14 (29%), grade 3–4 in 3 (6%) (Fig. 1C)). Neutropenia and anaemia were seen in 12 (24%) and 9 (18%), respectively, with grade 3–4 in 5 (10%) and 4 (8%), respectively. Haematological toxicity was the reason for treatment discontinuation in 2 patients, both for grade 3 anaemia.

Twenty-five (51%) patients experienced non-haematological toxicities. Whilst these were mild and tolerable without dose modification in 9 (18%) patients, 11 (22%) required dose reductions and 5 (10%) stopped treatment because of non-haematological toxicity. The commonest toxicities were fatigue (11, 22%), fluid retention (6, 12%), bone pain (5, 10%), and nausea (5, 10%) (Fig. 1D). In those stopping asciminib because of toxicity, often multiple toxicities were present concomitantly. Pericardial and pleural effusions were seen in 1 patient each, and successfully managed with dose reductions.

One patient suffered a myocardial infarction after 4 months of treatment, one a TIA after 24 months, and one a recurrence of a deep venous thrombosis (DVT). In all three patients a causal association was not clear because other risk factors co-existed.

No significant differences were seen in the frequency of toxicities (haematological or non-haematological) between those treated with standard (40 mg twice daily) or escalated doses (>40 mg twice daily), with haematological toxicity rates of 41% vs. 30%, respectively (*P* = 0.523) and non-haematological 49% vs. 60%, (*P* = 0.524), respectively.

### Response

Forty-four patients were considered eligible for response assessment after excluding 4 patients who stopped early for toxicity, before meaningful response assessment (median duration of treatment prior to stopping 1 [<1–2] month(s)), and 1 for poor compliance.

With a median duration of treatment of 14 [2–60] months in the response cohort, 29 (66%) patients had achieved or maintained a CCyR or better, with most patients (*n* = 26, 59%) achieving MMR or better (Fig. 1B). While most patients (*n* = 32, 73%) were not in CCyR prior to starting treatment with asciminib, all of those who were (*n* = 12, 27%) maintained or deepened their response on asciminib (supplemental Fig. 2).

Factors associated with higher rates of CCyR were intolerance rather than resistance to the last-line of therapy (86% vs. 31%, *P* < 0.001), and attainment of CCyR to any prior-line of therapy (84% vs. 17%, *P* < 0.001) (Table 2). Of those patients who had received prior ponatinib treatment, higher rates of CCyR were also seen in those who stopped for intolerance rather than resistance (71% vs. 25%, *P* = 0.032). There were no statistically significant differences in the rates of CCyR between those with or without a history of a T315I-BSNV (56% vs. 69%, *P* = 0.463), however it is important to note that all those with a T315I-BSNV tolerated escalated dose schedules. A history of a non-T315I BSNV was associated with a significantly lower rate of CCyR (42% vs. 75%, *P* = 0.038).

**Table 2:** Factors associated with achievement of complete cytogenetic response (CCyR) (restricted to 44 patients eligible for response assessment).

Characteristic		Number achieving CCyR or better (%)	*p*-value univariate 2-sided pearson ch-sq
**Reason for cessation of last-line of therapy**			
	Resistance (*n* = 16)	5 (31%)	<0.001
	Intolerance (*n* = 28)	24 (86%)	
**Prior response to any TKI (CCyR)**			
	Achieved CCyR (*n* = 32)	27 (84%)	<0.001
	No prior CCyR (*n* = 12)	2 (17%)	
**Prior ponatinib use**			
	No prior ponatinib (*n* = 19)	15 (79%)	0.112
	Prior ponatinib (*n* = 25)	14 (56%)	
**Prior ponatinib response**			
	Ponatinib intolerant (*n* = 17)	12 (71%)	0.032
	Ponatinib resistant (*n* = 8)	2 (25%)	
**History of any prior BSNV**			
	No prior BSNV (*n* = 25)	19 (76%)	0.105
	Prior BSNV (*n* = 19)	10 (53%)	
**Prior history of T315I-BSNV**			
	No prior T315I BSNV (*n* = 35)	24 (69%)	0.463
	Prior T315I BSNV (*n* = 9)	5 (56%)	
**Prior history non-T315I-BSNV**			
	No prior non-T315I BSNV (*n* = 32)	24 (75%)	0.038
	Prior non-T315I BSNV (*n* = 12)	5 (42%)	

*CCyR* complete cytogenetic response, *Ch-sq* chi-squared, *TKI* tyrosine kinase inhibitor, *BSNV*, *BCR::ABL1* single nucleotide variants.

### BSNV screening by NGS

**Fig. 2:**
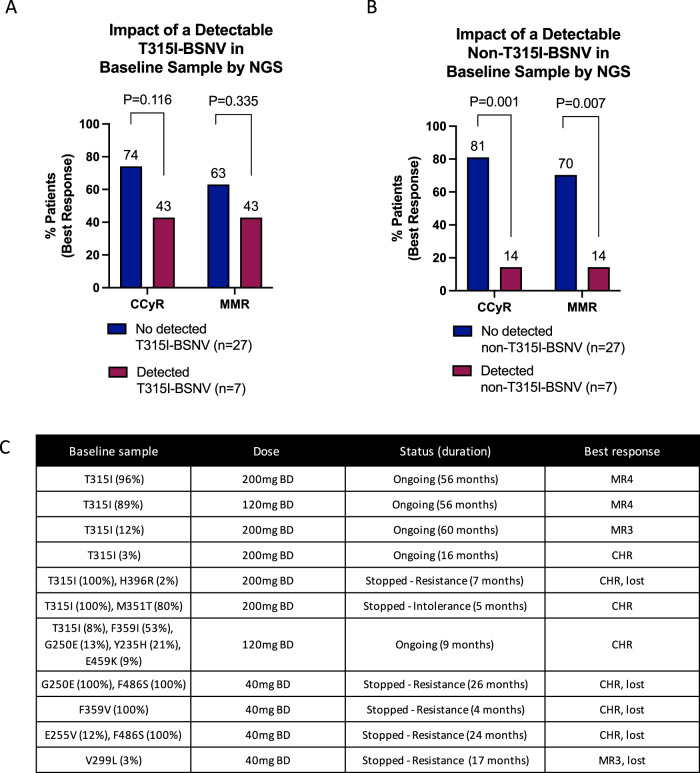
Impact of *BCR::ABL1* single nucleotide variants (BSNVs) detected in baseline samples on response to asciminib treatment. **A** impact of detectable T315I-BSNV in baseline sample on best response achieved, **B** impact of detectable non-T315I-BSNV in baseline sample on best response achieved, **C** details of BSNV detected in baseline samples with dose, treatment status, and best response information. BSNV, *BCR::ABL1* single nucleotide variants, NGS: next generation sequencing, mg: milligrams, CHR: complete haematological response, CCyR: complete cytogenetic response (*BCR::ABL1* IS level ≤ 1%), MMR: major molecular response (*BCR::ABL1* IS level ≤ 0.1%), BD: bis in die (twice a day).

Baseline samples were screened for BSNV by NGS in 34 patients, with paired samples at the time of stopping in 10 (8 stopped for resistance, 2 stopped for intolerance), and in samples during ongoing treatment in 16 (Fig. 2, Supplementary Fig. 1). All patients with BSNV screening by NGS were deemed eligible for response assessment.

### Baseline BSNV and response to asciminib

Nineteen BSNVs were detected in baseline samples of 11 (32%) patients prior to asciminib initiation. These were T315I alone in 4 patients, non-T315I-BSNV alone in 4, and combined T315I and non-T315I in 3. Five (45%) of 11 patients with baseline BSNV continued on treatment at the time of assessment compared to 17 (74%) of 23 patients without BSNV (*P* = 0.130). The reasons for stopping in those with a BSNV were resistance in 5 (45%) and intolerance associated with a poor response (*BCR::ABL1* IS level of 14% at 5 months of treatment) in one (9%), compared to resistance in 3 (13%), intolerance in 1 (4%), TFR and attempted pregnancy in DMR in 1 (4%) each, in those without BSNV.

Only 4 of 11 (36%) patients with a baseline BSNV achieved CCyR compared to 19 of 23 (83%) without (*P* = 0.007), with median follow-ups of 16 [4–60] months and 15 [4–44] months, respectively. This difference was most striking in those with non-T315I-BSNV. While there was no statistically significant difference in CCyR rates between those with or without a detectable T315I-BSNV in the baseline sample (43% vs. 74%, p0.116, median duration of treatment 9 [5–60] vs. 16 [4–44] months), only 1 of 7 (14%) patients with a detectable non-T315I-BSNV achieved CCyR, compared to 22 of 27 (81%) without (*P* = 0.001, Fig. 2A–C, median durations of treatment 9 [4–26] months vs. 16 [4–60] months).

### BSNV presence at time of cessation

**Fig. 3:**
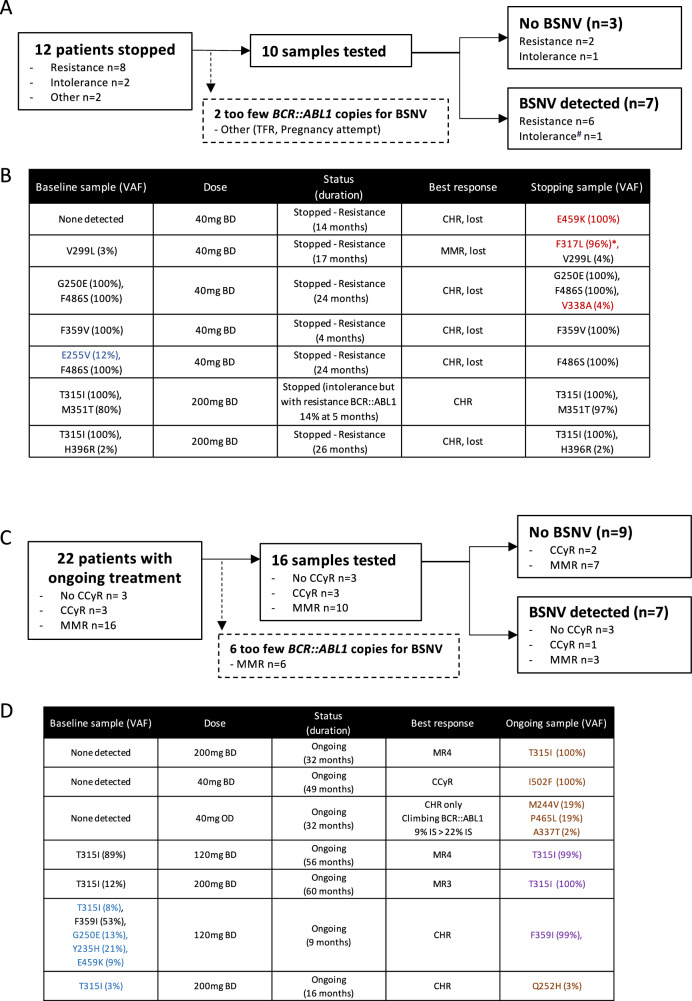
Detection of *BCR::ABL1* single nucleotide variants (BSNVs) in final (stopping) and most recent (ongoing) samples. **A** schematic of stopping samples analysed, **B** details of BSNV in baseline sample, asciminib dose, treatment status, best response, BSNV detected in the sample at the time of stopping, BSNV denoted in red are new in the stopping samples, those in blue are no longer detectable in the stopping sample, **C** schematic of ongoing treatment samples analysed, **D** details of BSNV in baseline sample, asciminib dose, treatment status, best response, BSNV detected in the most recent sample while on treatment, BSNV denoted in orange are new in the treatment samples, those in blue in the baseline sample are no longer detectable in the treatment sample, those in purple have expanded on treatment. BSNV, *BCR::ABL1* single nucleotide variant, CHR: complete haematological response, CCyR: complete cytogenetic response (*BCR::ABL1* IS level ≤ 1%), MMR: major molecular response (*BCR::ABL1* IS level ≤ 0.1%), BD: bis in die, twice a day, OD: once daily, IS, international scale. ^#^stopped treatment for intolerance associated with poor response, *total F317L amino acid substitution from both c.949 T > C (45%) and c.951 C > A (51%).

Thirteen BSNVs were detected in 7 patients at the time of stopping (Fig. 3A, B), 6 of whom stopped treatment for resistance, and one for intolerance with an associated poor response (*BCR::ABL1* IS level of 14% at 5 months of treatment). In 4 patients the BSNVs were present at baseline, of which two had no significant change in the VAF during treatment: a single F359V (VAF 100% baseline and stopping) and a compound T315I (VAF 100% baseline and stopping) with H396R (minor clone, VAF 2% at baseline and stopping). In one patient, expansion of a compound clone (T315I/M351T) was seen, (80% at baseline and 97% at cessation), and one patient had loss of a small subclone with persistence of a larger clone carrying only 1 BSNV (F486S, VAF 100% and E255V, VAF 12% at initiation, and F486S, VAF 100% only at stopping). Three patients had emergent BSNV that were not present in the baseline samples. One patient showed the re-emergence of a historically noted E459K-BSNV that was undetectable at initiation, but 100% VAF at the time of loss of response and treatment cessation. One patient who had achieved MMR, but subsequently lost response, showed the emergence of two BSNVs (c.949 T > C p.F317L, VAF 45% and c.951 C > A p.F317L, VAF 51%), both of which resulted in the same amino acid substitution (F317L) but in independent clones (Supplementary Fig. 3), with VAFs totalling 96%, in combination with a low-level V299L (VAF 4%), which had been present in the baseline sample (baseline VAF 2%). The remaining patient showed the emergence of a low-level BSNV (V338A, VAF 4%) in association with high VAF compound BSNV G250E/F486S (VAFs 100% at baseline and stopping), most likely a subclone carrying 3 BSNVs, associated with loss of haematological response and transformation to blast phase.

### BSNV during ongoing treatment

BSNVs screening was possible in 16 out of 22 patients who continue on treatment (6 had insufficient *BCR::ABL1* copy numbers for NGS). Of these 16, 3 had achieved haematological response only, 3 CCyR and 10 MMR or deeper. Nine BSNV were detected in 7 patients (Fig. 3C,D). Of these, one patient had the emergence of a novel BSNV (I502F), not present in the baseline sample or historical records. It represented the dominant clone (VAF 82%), and was seen in the context of standard asciminib dosing, achieving a best response to the treatment of CCyR.

In one patient receiving high dose asciminib because of a history of a T315I-BSNV, an F359I-BSNV, which was present at initiation along with other BSNVs (T315I, VAF 8%, G250E, VAF 13%, Y235H, VAF 21% and E459K, VAF 9%) emerged as the sole mutation in a dominant clone (VAF 53% at asciminib initiation and subsequently 99%, Fig. 3D). Three BSNVs (M244V, VAF 19%, P465L, VAF 19% and A337T, VAF 2%) were emergent in a patient achieving haematological response only. In one patient receiving high dose asciminib because of a history of a T315I-BSNV there was loss of the low-level T315I-BSNV, and an emergent Q252H BSNV at low-level (VAF 3%).

Interestingly, three patients had detectable T315I mutations, with VAFs consistent with their presence in the dominant clone in most recent sample on treatment. All three were seen in patients with a known history of T315I mutations, two of whom were tolerating maximal dose asciminib (200 mg BD), one achieving MMR and one MR4, while the remaining patient had achieved MMR on 120 mg BD (dose reduced for toxicity). While the T315I mutation had been detectable in the baseline sample in 2, it was undetectable in one patient suggesting subsequent clonal selection of a very low-level T315I-harbouring clone.

### BSNV dynamics over time

**Fig. 4:**
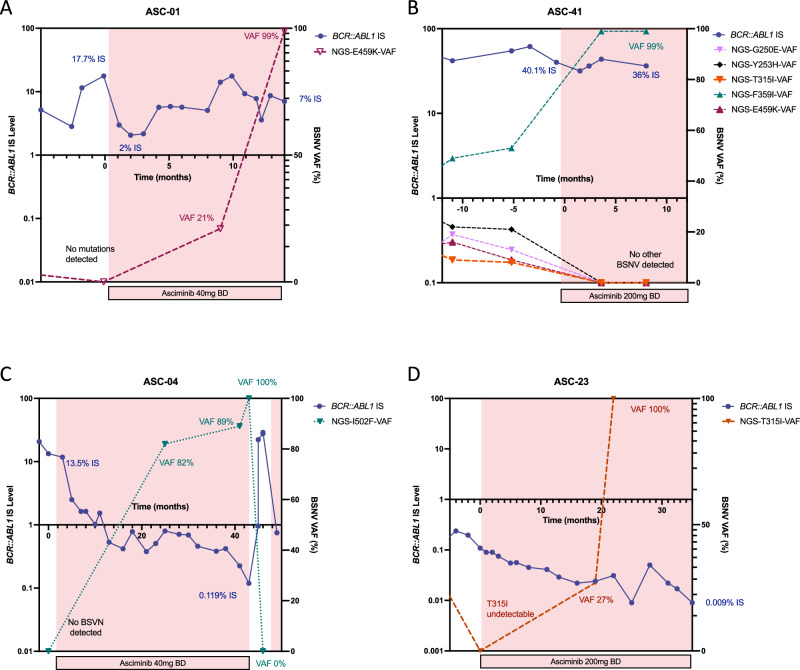
*BCR::ABL1* single nucleotide variant dynamics over time. **A** patient ASC-01, *BCR::ABL1* IS level (blue) and E459K-BSNV VAF (purple) over time, starting before (white background), then during treatment with asciminib (pink background), until asciminib cessation, **B** patient ASC-41, *BCR::ABL1* IS level (blue), and G250E- (lavender), Y253H- (black), T315I- (orange), F359I- (green), E459K- (maroon) BSNV VAF over time, before (white background) and during (pink background) asciminib treatment **C** patient ASC-04, *BCR::ABL1* IS level results (blue) and I502F-BSNV VAF (teal) before (white background) and during (pink background) asciminib treatment, **D** patient ASC-23, *BCR::ABL1* IS level (blue) and T315I-BSNV VAF (orange) before (white background) and during (pink background) asciminib treatment. *BCR::ABL1* IS level (left y-axis), BSNV expressed as variant allele frequency (right y-axis) over time (months, x-axis), BSNV, *BCR::ABL1* single nucleotide variant, VAF, variant allele frequency, BD, bis in die, twice a day, IS, international scale.

Four patients with BSNV were selected for additional NGS-BSNV screening at timepoints before, during and after asciminib treatment to better understand the clonal dynamics. The first patient (ASC-01, Fig. 4A) had received 4 prior TKIs, most recently ponatinib which had achieved MMR, but was stopped for intolerance. He had a known history of an E459K-BSNV detected by Sanger sequencing during a prior-line of therapy, however the BSNV screen by NGS at initiation of asciminib was negative. While there was an initial decline in the *BCR::ABL1* IS level from 17.7% to 2% upon treatment with asciminib 40 mg twice daily, this was not sustained. Loss of response with a rising *BCR::ABL1* IS level, followed by loss of haematological response was associated with clonal expansion of the E459K-harbouring clone with a VAF of 21%, then 100%, indicative of clonal selection of the E459K-BSNV-harbouring population. Retrospective sample sequencing showed that this pattern of clonal selection for the E459K-BSNV population was not seen during prior ponatinib treatment (Supplementary Fig. 4A).

The second patient (ASC-41, Fig. 4B) had received 5 prior TKIs and had most recently stopped ponatinib for primary resistance. Given a known history of multiple BSNVs, including a T315I (M244V, G250E, Y253H, T315I, M351Y, F359I, H396R, S438C and E459K), the patient received asciminib 200 mg twice daily. Prior to initiation of asciminib, BSNV analysis showed the presence of G250E (VAF 13%), Y253H (VAF 21%), T315I (VAF 3%), F359I (VAF 53%) and E459K (VAF 9%)-BSNVs, without evidence of the historically noted M244V, M351Y, H396R or S438C. While no significant reduction was seen in the *BCR::ABL* IS level, there was a change in the landscape of BSNV-containing clones with the emergence of a dominant clone carrying an isolated F359I-BSNV. The additional BSNVs, including the E459K, were no longer detectable, which may indicate that the clonal selection of E459K harbouring cells seen in patient ASC-01 on asciminib 40 mg BD may be overcome with intensified dosing.

The third patient (ASC-04, Fig. 4C) had received 4 prior TKIs, most recently bosutinib which was stopped for intolerance, and had previously achieved an MMR on dasatinib and nilotinib subsequently stopped for intolerance. They had no history of BSNV despite multiple screens by Sanger sequencing. Following initiation of asciminib the patient had a steady decline in their *BCR::ABL1* IS level from 13.5% IS to a nadir of 0.119% IS, but never achieved MMR. During treatment with asciminib 40 mg twice daily, an emergent clone with an I502F-BSNV became dominant, with a progressive rise in VAF to 82%, then 89% and finally 100% on subsequent samples despite maintained CCyR. Interestingly upon treatment pause (during an episode of heart failure subsequently deemed unrelated to asciminib) there was a rapid rise in *BCR::ABL1* IS level, with a fall in the VAF of I502F-BSNV to below the level of detection, indicating relative outgrowth of I502F harbouring cells by the non-mutated BCR::ABL1 population. Collectively this suggests that asciminib exerts selective pressure on the I502F harbouring cells, conferring a degree of resistance, but nevertheless sufficient sensitivity to maintain a CCyR.

The fourth patient (ASC-23, Fig. 4D) had shown primary resistance to imatinib with an emergent T315I-BSNV detected by Sanger sequencing. Despite achieving CCyR on ponatinib, they developed intolerable toxicity, and changed to asciminib 200 mg BD. On asciminib, they continued to deepen their *BCR::ABL1* response, achieving MR4. Interestingly, the T315I mutation, that was below the level of detection by NGS at asciminib initiation, re-emerged with a rising VAF to 27%, then 100% during treatment, demonstrating the selective pressure exerted on T315I-harbouring cells by asciminib. Intriguingly, retrospective BSNV screening during the period of treatment with ponatinib does not show any evidence of clonal selection for T315I harbouring cells in that context (supplemental Fig. 4B).

## Discussion

Asciminib has demonstratable efficacy and tolerability in early- and late-phase clinical trials [8, 9, 19], and here we confirm these findings in real world data. In particular, the tolerability in pre-treated patients, often with multiple previous intolerances, is confirmed by the high rates of treatment continuation, corroborating that of other managed access programmes [20–23]. In addition, we report better responses in those who had achieved CCyR to any line of prior therapy, stopped their last-line of treatment for intolerance rather than resistance, and those with no history of non-T315I-BSNVs.

While we found no significant differences in response to asciminib in those with a prior history of a T315I-BSNV, with the caveat that these patients received high dose asciminib, a history of a non-T315I-BSNV, and in particular, the presence of a detectable non-T315I-BNSV at the time of initiation of asciminib was association with a lower rate of attainment of CCyR. Several BSNV have been shown to impact asciminib efficacy in vitro, specifically those close to the myristoyl site (e.g. A337V, P465S, V468F and I502L) [1, 24] and to a lesser extent, those in the SH2/kinase domain interface (e.g. P223S, K294E) [1], as well as some in the kinase domain such F359V/I, which shows extremely high IC50 values [12]. Some of these function by directly inhibiting asciminib binding (e.g. F359V), while others impact the conformational response to asciminib (e.g. M244V) [25]. Whilst many mutations tested in vitro show some degree of resistance to asciminib [1, 11], the true clinical relevance of this in vivo remains less clear, and crucially, whether these can be overcome with increased dosing, as in the case of T315I is broadly unknown.

Using NGS to track clonal dynamics we have also shown the emergence and/or expansion of BSNV-harbouring clones during asciminib treatment. In particular we show clear emergence of E459K, F317L and F359I clones that dominate the *BCR::ABL1*-positive population associated with loss of response or primary resistance. Importantly, however in one patient receiving high dose asciminib, we see positive selection/outgrowth of F359I-harbouing populations, with negative selection of, E459K- and T315I-harbouring populations. This may suggest that higher doses of asciminib can overcome the relative resistance conferred by the E459K-, and T315I-BSNV, but not the F359I-BSNV, and would corroborate the in vitro IC50s of 0.61 nM, 3.01 nM, 7.6 nM and 11.5 nM for non-mutated, E459K, T315I and F359V, respectively [11]. In addition, we see clonal selection and poor response to high dose asciminib in the setting of compound BSNVs (T315I/M351T), as well as resistance to standard dose with persisting dominant clones (compound G250E/F486S, F359V, F486S).

Interestingly, we show a pattern of clonal expansion and dominance in some patients with T315I-, and I502F-BSNVs, despite molecular responses in the *BCR::ABL1* IS level. Whilst not ubiquitous, this is an intriguing observation, and supports the notion that BSNV-mediated resistance in the setting of asciminib is less binary and, for some BSNV, the degree of resistance may be sufficient to promote clonal selection, but insufficient to result in treatment resistance or failure.

Whilst our data has several limitations, namely the retrospective nature of the data collection, and the ad hoc availability of samples for BSNV analysis by NGS, we can make some important observations, and provide valuable insight into clonal dynamics of BSNV-harbouring cells during treatment with asciminib. Whilst we cannot fully exclude BSNV-independent mechanisms of resistance, we show clear evidence of clonal expansion under the selective pressure of asciminib; but with varying degrees of clinical resistance (summarised in Table 3). Whilst some BSNV are associated with marked resistance even to high dose asciminib (F359I), the clonal dynamics suggests some may warrant further exploration of dose escalation in non-responders.

**Table 3:** Summary and interpretation of BSNV reported in this dataset.

Mutation	Evidence from this study	Published in vitro data	Published in vivo data#
Emerging BSNV-harbouring clones, associated with resistance. Suggests clonal selection associated with resistance. Likely clinically relevant.			
**E459K**	Positive clonal selection associated with treatment resistance in 1 patient receiving 80 mg daily, but negative selection against E459K-harbouring clone in 1 patient receiving 240 mg daily.	Modestly increased IC50 ~ 5 fold [1, 11].	Present in baseline sample of 1 patient in phase 3 study (isolated E459K) who failed to achieve MMR at week 24 and discontinued treatment, no clonal dynamic reported [9].
**F317L**	Positive clonal selection associated with treatment resistance in 1 patient receiving 80 mg daily, no data at higher doses.	Increased IC50 ~ 20 to >300 fold [12, 25].	Identified in 3 patients in phase 3 study: Isolated F317L present at baseline in 1 patient who failed to MMR at week 24 and discontinued treatment; Present at baseline in combination/compound (F317L/L248V) in 1 patient who failed to achieve MMR at week 24 and discontinued treatment; Emergent combination/compound (F317L/E355G) in 1 patient who failed to achieve MMR at week 24 and discontinued treatment, no clonal dynamic reported [9]. Present in dominant clone (VAF 100% at initiation and VAF 99% at follow-up) in 1 patient who achieved MMR, no dose information reported [12].
**F486S**	Positive clonal selection associated with treatment resistance in 1 patient receiving 80 mg daily, no data at higher doses.	No data.	No data on isolated F486S. Present in baseline sample in combination/compound with Y253H in 1 patient in phase 3 study who failed MMR at week 24 and discontinued treatment, no clonal dynamics reported [9].
**F359I**	Positive clonal selection associated with treatment resistance in 1 patient receiving 240 mg daily, no data at higher doses.	Increased IC50 > 500 fold [12].	Clonal selection for dominant clone harbouring isolated F359I in one patient (undetectable at baseline to VAF 99%) and F359V in one patient (VAF 79% at baseline to 98%) emergent from background populations harbouring multiple mutations, associated with treatment failure in both patients receiving doses up to 400 mg daily. Clonal selection in association with other BSNV in 1 patient (T315I, VAF 29% alone at baseline to F359I, VAF 46%, T315I, VAF 40%, A433D, VAF 11%, P112S, VAF 3%) [12]. Isolated F359C and F359V in baseline samples of 1 and 3 patients respectively in phase 3 study, all of whom failed to achieve MMR at 24 weeks and discontinued treatment, no clonal dynamics reported [9]. F359V present in 1 patient on compassionate use programme who failed to achieve CCyR (80 mg daily), no clonal dynamics reported [22].
**Compound T315I/M351T**	Positive clonal selection associated with treatment resistance in 1 patient receiving 400 mg daily.	Increased IC50 > 500 fold [12].	No data.
**BSNV stably present in a dominant clone, associated with resistance**. May represent BSNV-mediated or non-BSNV mediated mechanism of resistance with co-existing BSNV passenger mutations. *Possibly clinically relevant*.			
**F359V**	Resistance with ongoing clonal dominance in 1 patient receiving 80 mg daily.	Increased IC50, ~20- to >500-fold [1, 11, 12, 25].	Refer to F359I above.
**Compound G250E/F486S**	Resistance with ongoing clonal dominance in 1 patient receiving 80 mg daily.	No compound data; isolated G250E modestly increased IC50 ~ 1.5- to 5-fold [12, 25]; isolated F486S no data.	No data on compound mutations. See F486S above and G250E below for isolated mutation data.
**T315I with low-level H396R**	Resistance with unchanged clone size in 1 patient receiving 400 mg daily.	Increased IC50 > 500-fold in compound [12]; Isolated T315I increased IC50 ~ 8–12-fold [1, 11, 12]; Isolated H396R increased IC50 ~ 16-fold [12].	No data on compound mutations. Established resistance of T315I alone to standard doses, clinical responses achieved on 200 mg BD [8, 13]. Evidence of clonal selection on standard dose, overcome with higher dose in non-responding patients [12]. No data on isolated H396R.
**Emerging BSNV-harbouring clones with ongoing clinically meaningful response**. Indicates clonal selection, but insufficient resistance to preclude clinical response. *Unknown clinical relevance*.			
**I502F**	Positive clonal selection in 1 patient receiving 80 mg daily, who achieved sustained CCyR.	No data on I502F; Increased IC50 for I502L ~ 50-fold [1].	No data on I502F. Clinical progression in 1 patient in phase 1 study, in AP associated with emergence of I502L (VAF 87.8%), V468F (VAF 11.9%) and E355G (VAF 1.1%) [8].
**T315I**	Positive clonal selection at 400 mg daily, but achieved deep molecular responses in 3 patients.	Increased IC50 ~ 8-12-fold [1, 11, 12, 25].	Established resistance of T315I to standard doses, clinical responses achieved on 200 mg BD [8, 13]. Evidence of clonal selection on standard dose, overcome with higher dose in non-responding patients [12].
**Low-level variants associated with resistance**. Potentially passenger BSNV in the context of non-BSNV-mediated resistance. *Unknown clinical relevance*.			
**Q252H**	Emergence at low-level (3%) associated with treatment resistance in 1 patient receiving 400 mg daily.	Increased IC50 ~ 4-18-fold [1, 11, 12].	No data.
**V338A**	Emergence at low-level (4%) associated with treatment resistance in 1 patient receiving 80 mg daily.	No data.	No data.
**V299L**	Persistence of low-level clone associated with treatment resistance in 1 patient receiving 80 mg daily (3% at imitation, 4% at cessation)	Modestly increased IC50 ~ 10-fold [1].	No data in chronic phase. Present in 1 patient on in phase 1 study, in AP treated with 40 mg BD who progressed, without evidence of clonal selections/dominance [8].
**BSNV-harbouring clones negatively selected against**. Suggestive of sensitivity to asciminib. *Possibly clinically relevant*.			
E255V*	Negatively selected against despite treatment resistance in 1 patient receiving 80 mg daily.	Modestly increased IC50 ~ 3–4-fold [1, 11, 12].	E255V and E255K present in baseline samples in 1 and 2 patients respectively in phase 3 study who achieved MMR at week 24 and continued treatment, no clonal dynamics reported [9]. E255K present in dominant clone (VAF 100%) at baseline in 1 patient in phase 1 study who achieved MMR on 40 mg BD but subsequently lost response with emergence of G463S mutation (VAF not reported) [8]. E255K presented in combination with T315I in 1 patient on compassionate use programme who failed to achieve a response on 400 mg daily, no clonal dynamics reported [21].
G250E*	Negatively selected against despite treatment resistance in 1 patient receiving 240 mg daily.	Modestly increased IC50 ~ 5–6-fold [12, 25].	Isolated G250E in baseline sample of 1 patient in phase 3 study who failed to achieve MMR at week 24 but continued treatment, and emergent in 1 patient in phase 3 study who achieved MMR at week 24 and continued treatment, no clonal dynamics reported [9]. Present in 1 patient on compassionate use programme achieving MR4 receiving 80 mg daily, no clonal dynamics reported [22].
Y253H*	Negatively selected against despite treatment resistance in 1 patient receiving 240 mg daily.	Modestly increased IC50 ~ 2–6-fold [1, 11, 12, 25].	Isolated Y253H in baseline sample of 1 patient who achieved MMR at week 24 and continued treatment, and emergent in 1 patient in who failed to achieve MMR at week 24 and discontinued treatment in phase 3 study, no clonal dynamics reported [9]. Isolated Y253H in 1 patient on compassionate use programme achieving CCyR on 80 mg daily, use no clonal dynamics reported [22]. Present in combination with T315I in 1 patient on compassionate use programme with AP who failed to achieve a response on 200 mg BD [21].
BSNV identified in the study, insufficient data to classify. *Unable to classify*.			
M244V*,**	Positive clonal selection in combination with P465L and A337T in 1 patient with haematological response only.	Increased IC50 ~ 20-fold [25]	Associated with treatment resistance and emergence/clonal dominance in 4 patients, some receiving up to 200 mg BD [25]. Present in baseline sample of 1 patient in phase 1 study who developed progressive disease on treatment, no dose or clonal dynamics reported [8]. Present as isolated M244V in 1 patient who failed to achieve CCyR on 80 mg daily, and in combination with F317L and E255K in 2 further patients, one in AP who failed to achieve CCyR on 200 mg daily, and one in CP who achieved CCyR on 80 mg daily, on compassionate use programme, no clonal dynamics reported [22]. Present in combination with a F317L in 1 patient on compassionate use programme achieving CCyR on 40 mg BD, no clonal dynamics reported [21]
P465L*,**	Positive clonal selection in combination with M244V and A337T in 1 patient with haematological response only.	No data in P465L; P465S associated with increased IC50 > 500-fold [12]	No data on P465L; Emergent P465S mutation (VAF8%) in combination with G109D (VAF3.3%) and T315I (VAF100%) in 1 patient in phase 1 study on asciminib treatment with up to 200 mg BD and no notable response [8].
A337T*,**	Positive clonal selection at low level (VAF 3%) in combination with M244V and P465L in 1 patient with haematological response only.	No data in A337T; A337V increased IC50 > 500-fold [1, 12]	Emergent A337T mutation (VAF 37%*)* in combination with G463D (VAF 8.7%) and Y115N (VAF 5%) with re-existing T315I (VAF 40.4%) in 1 patient on up to 200 mg BD in phase 1 study [8]. Present at low level without evidence of clonal selection patients in doses up to 200 mg BD [12]

BSNV, *BCR::ABL1* single nucleotide variant, *mg* milligrams, *IC50* half maximal inhibitory concentration, *CCyR* complete cytogenetic response (*BCR::ABL1* IS level ≤ 1%), *MMR* major molecular response (*BCR::ABL1* IS level ≤ 0.1%), MR4, *BCR::ABL1* IS level ≤ 0.01% IS, *VAF* variant allele fraction, *CP* chronic phase, *AP* accelerated phase, *IS* international scale. * Reported in the context of more than a BSNV, unable to exclude compound clones/BSNV interaction, ** Unable to differentiate co-existing or compound state (M244V (19%), P465L (19%), A337T (3%)), **±** Note that IC50 > 1 but ≤10 fold more than non-mutant BCR::ABL1 have been defined as modestly increased, IC50 > 10 are defined as increased, # Unless otherwise stated, in vivo data reports to chronic phase patients receiving standard dose regimens (80 mg daily).

Whilst the observation that T315I harbouring cells dominate the BCR::ABL1-positive population in some patients during treatment is intriguing, and supports the notion that asciminib exerts a degree of selective pressure in this setting, it should not be over-interpreted. In this cohort we have performed BSNV screening in patients in whom it would not typically have been considered (i.e. responding patients, with low *BCR::ABL1* IS levels) [18], therefore the data to put this finding into context are sparse. Nevertheless, these data raise questions of whether this is a typical phenomenon in patients with BSNV treated with other TKI to which they are sensitive, or is a phenomenon unique to asciminib.

### Data sharing statements

Original data are available to those with reasonable requests, and appropriate ethical approval, by contacting the corresponding author.

## Supplementary information

Supplemental Material
