# Supplementary material for: Impact of BCR::ABL1 single nucleotide variants on asciminib efficacy
Source: Leukemia. 2024 Sep 17;38(11):2443–55. doi: 10.1038/s41375-024-02411-7 (PMC11518997; doi:10.1038/s41375-024-02411-7)
Supplement: Supplementary file 1 — Supplemental Material [file 41375_2024_2411_MOESM1_ESM.pdf]

Supplemental methods:

BCR::ABL1 mutation screening was performed by next generation sequencing after amplification of *BCR::ABL1* transcripts with a forward primers either in exon 1 or exon 13 BCR and reverse primer in exon 10 ABL1. For some samples a nested PCR was performed using the same set of primers. PCR product quality check was performed on the Qiaxcel analyser and quantified using Qubit™ dsDNA broad range assay kit (Life Technologies). Tagmentation of the PCR product was performed using the Nextera XT DNA library preparation kit (Illumina) (1ng DNA) and limited-cycle PCR to add index adapter sequences. Libraries were purified using the Agencourt AMPure XP reagent (Beckman Coulter) [bead technology], quality checked and quantified using Qubit™ dsDNA high sensitivity assay kit (Life Technologies). 10pM of library was loaded into the MiSeq v2 Nano reagent cartridge (500 cycles) (Illumina) and sequenced using 151 base paired end runs. Sequencing alignment and somatic variant calling was performed using the preinstalled local run manager software v2.0 using the PCR amplicon workflow and a custom manifest aligned to reference sequence NM_005157.6. The mean read depth was 12000 reads. Integrative Genomics Viewer v2.10.0 was used to visualise alignment and variants that met the following criteria were included: read depth >1,000 with a VAF >3%. Variants detected <3% that were reproducible across independent NGS runs were included, with a caveat of below LOD.

Supplemental table 1 – Historical TDKM list (full cohort, n=49)

Mutation(s)	Frequency (number of patients)
T315I alone	9
M244V; G250E; Y253H; T315I; M351Y; F359I; H396R; S438C; E459K	1
Y253H	1
E255V; T315I; M351T	1
E292G	1
V299L	1
V299L, F317L	1
V299L; F486S	1
F359V	1
E459K	1
E459K; M244V	1
G250; E462K; F486S	1
S438P	1

Supplemental table 2– Historical TDKM list (restricted to response assessment cohort, n=44)

Mutation(s)	Frequency (number of patients)
T315I alone	7
M244V; G250E; Y253H; T315I; M351Y; F359I; H396R; S438C; E459K	1
Y253H	1
E255V; T315I; M351T	1
E292G	1
V299L	1
V299L, F317L	1
V299L; F486S	1
F359V	1
E459K	1
E459K; M244V	1
G250; E462K; F486S	1
S438P	1

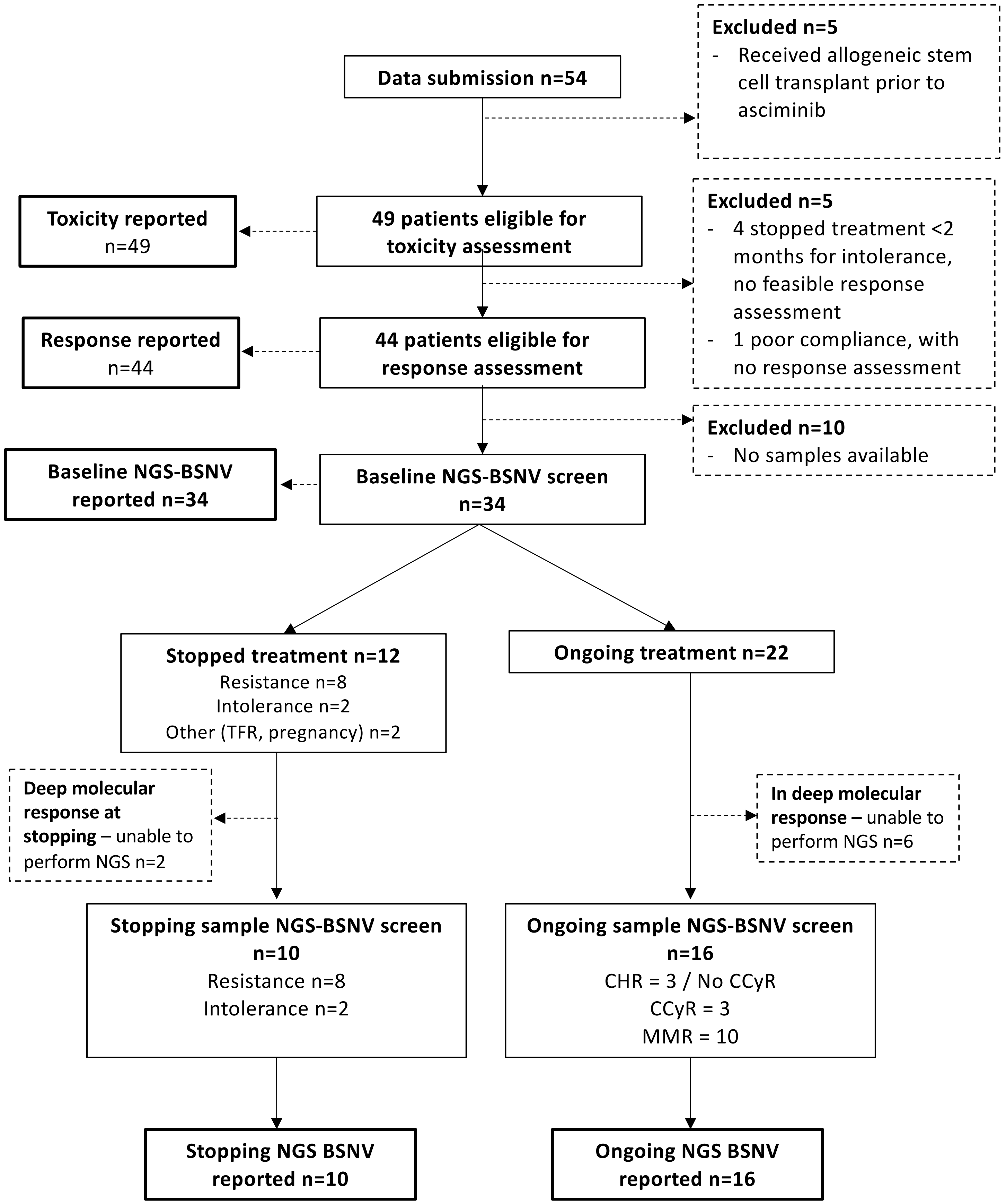

Supplemental figure 1 – Consort diagram, and next generation sequencing sample availability, NGS, next generation sequencing, BSNV, *BCR::ABL1* single nucleotide variants, CHR, complete haematological response, CCyR, complete cytogenetic response, MMR, major molecular response.

A

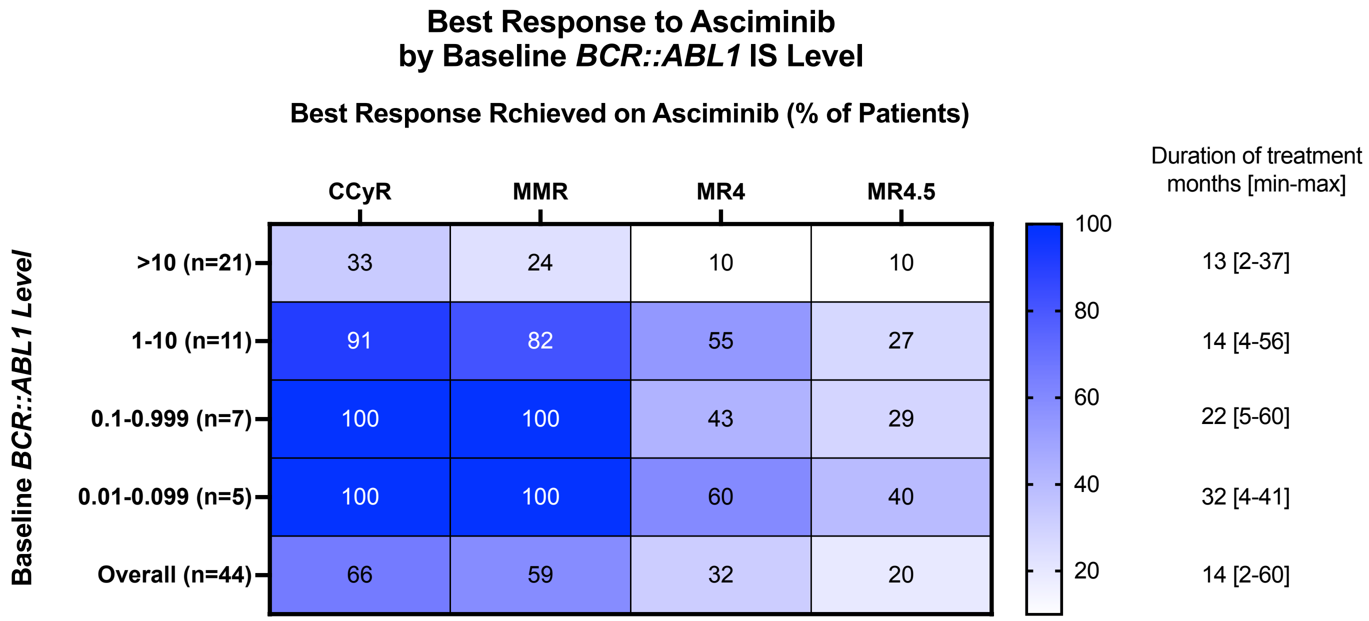

Supplemental figure 2 – Percentage of patients achieving response by baseline *BCR::ABL1* PCR. CCyR, complete cytogenetic response (*BCR::ABL1* IS level ≤ 1%), MMR, major molecular response (*BCR::ABL1* IS level ≤ 0.1%), MR4 (*BCR::ABL1* IS level ≤ 0.01%), MR4.5 (*BCR::ABL1* IS level ≤ 0.0032%).

A

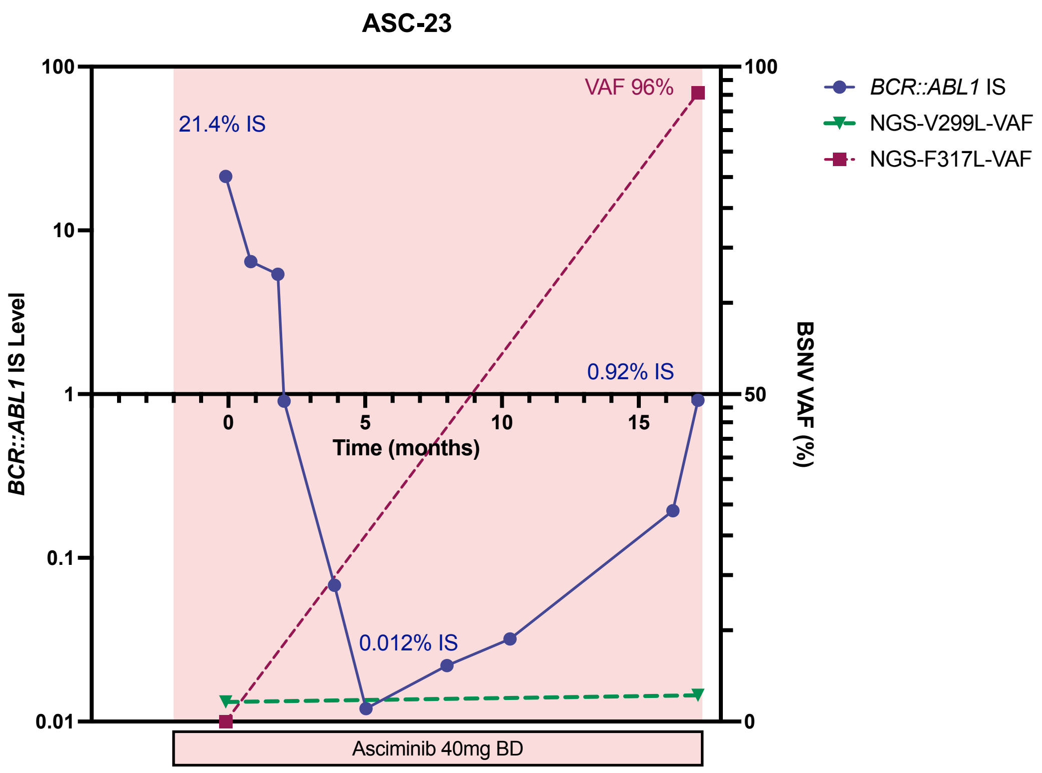

B

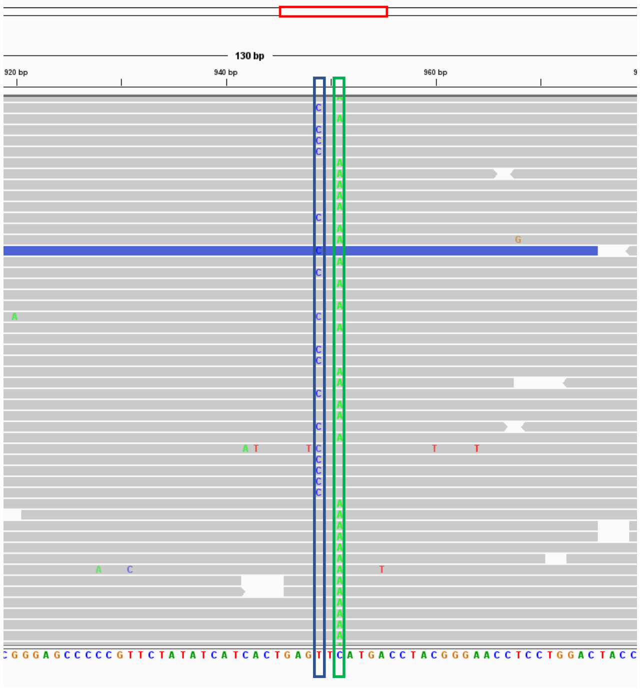

Supplemental figure 3 – a) BCR::ABL1 IS level and BSNV clonal dynamics during treatment with asciminib, *BCR::ABL1* IS level (blue), V299L-BSNV VAF (green) and total F317L-BSNV VAF (maroon), treatment period on asciminib denoted by pink background. b) sequencing reads of *BCR::ABL1* NGS. Blue box highlights c.949T>C substitutions coding p.Phe317Leu (F717L) and green box highlights c.951C>A substitution coding p.Phe317Leu (F317L) demonstrating that the sequence variants are mutually exclusive.

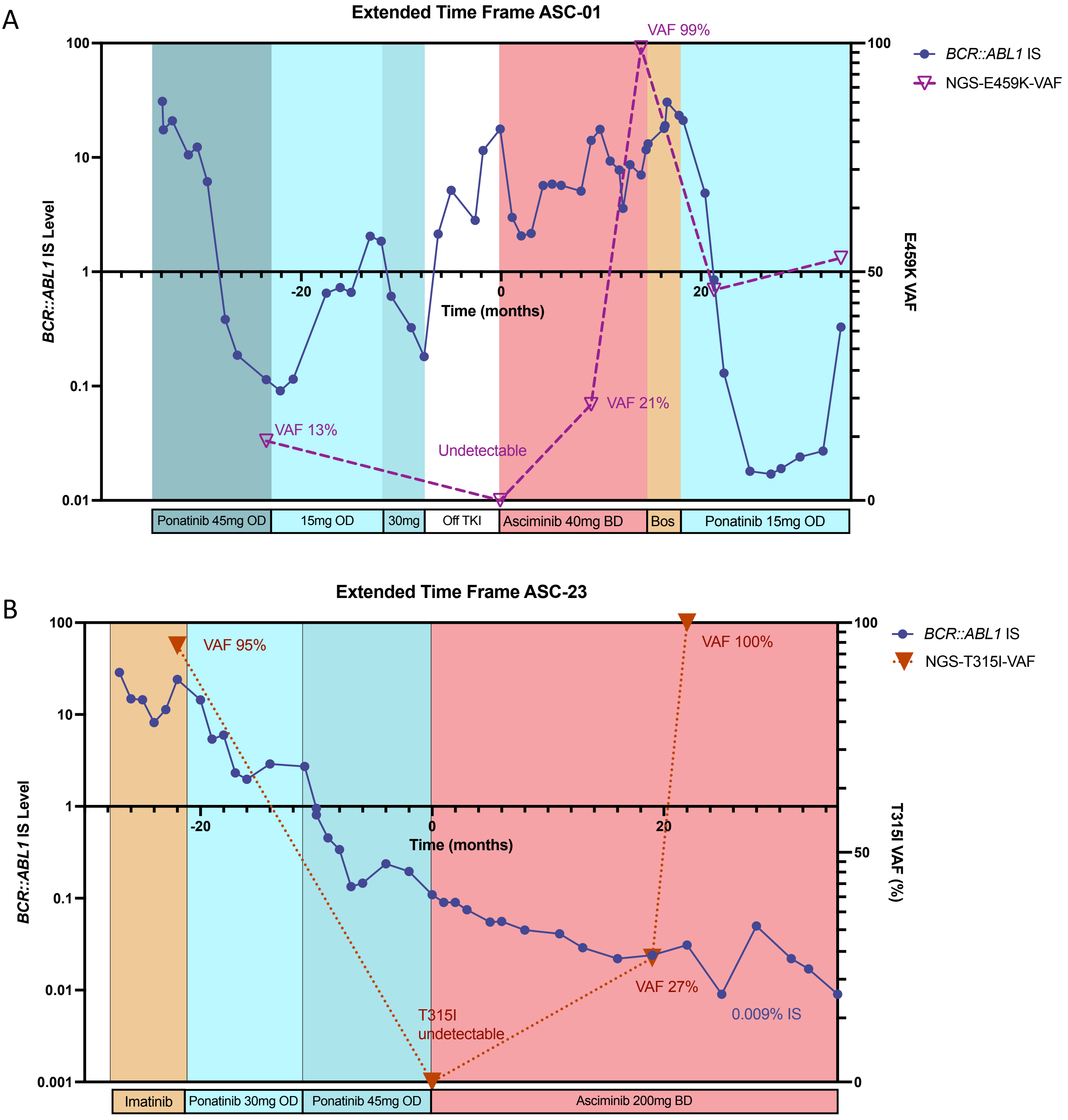

Supplemental Figure 4 – Extended timeframe *BCR::ABL1* IS level and BSNV VAF clonal dynamics from patient ASC-01, (figure 4a), *BCR::ABL1* IS ratio (blue) and E459K-BSNV VAF (purple) with treatments noted on the X axis and b) patient ASC-23, (figure 4d), *BCR::ABL1* IS ratio (blue) and T315I BSNV VAF (orange) , with treatments noted on the X axis. VAF, variant allele frequency.
